# Sharing Family Life Information Through Video Calls and Other Information and Communication Technologies and the Association With Family Well-Being: Population-Based Survey

**DOI:** 10.2196/mental.8139

**Published:** 2017-11-23

**Authors:** Chen Shen, Man Ping Wang, Joanna TW Chu, Alice Wan, Kasisomayajula Viswanath, Sophia Siu Chee Chan, Tai Hing Lam

**Affiliations:** ^1^ School of Public Health The University of Hong Kong Hong Kong China (Hong Kong); ^2^ School of Nursing The University of Hong Kong Hong Kong China (Hong Kong); ^3^ Center for Community-Based Research Dana-Farber Cancer Institute Harvard TH Chan School of Public Health Cambridge, MA United States; ^4^ Department of Social and Behavioral Sciences Harvard TH Chan School of Public Health Cambridge, MA United States

**Keywords:** mobile phone, video call, Chinese

## Abstract

**Background:**

The use of information and communication technologies (ICTs) for information sharing among family members is increasing dramatically. However, little is known about the associated factors and the influence on family well-being.

**Objective:**

The authors investigated the pattern and social determinants of family life information sharing with family and the associations of different methods of sharing with perceived family health, happiness, and harmony (3Hs) in Hong Kong, where mobile phone ownership and Internet access are among the most prevalent, easiest, and fastest in the world.

**Methods:**

A territory-wide population-based telephone survey was conducted from January to August 2016 on different methods of family life information (ie, information related to family communication, relationships with family members, emotion and stress management) sharing with family members, including face-to-face, phone, instant messaging (IM), social media sites, video calls, and email. Family well-being was assessed by three single items on perceived family health, happiness, and harmony, with higher scores indicating better family well-being. Adjusted prevalence ratios were used to assess the associations of sociodemographic factors with family life information sharing, and adjusted beta coefficients for family well-being.

**Results:**

Of 2017 respondents, face-to-face was the most common method to share family life information (74.45%, 1502/2017), followed by IM (40.86%, 824/2017), phone (28.10%, 567/2017), social media sites (11.91%, 240/2017), video calls (5.89%, 119/2017), and email (5.48%, 111/2017). Younger age and higher education were associated with the use of any (at least one) method, face-to-face, IM, and social media sites for sharing family life information (all *P* for trend <.01). Higher education was most strongly associated with the use of video calls (adjusted prevalence ratio=5.61, 95% CI 2.29-13.74). Higher household income was significantly associated with the use of any method, face-to-face, and IM (all *P* for trend <.05). Sharing family life information was associated with a higher level of perceived family well-being (beta=0.56, 95% CI 0.37-0.75), especially by face-to-face (beta=0.62, 95% CI 0.45-0.80) and video calls (beta=0.34, 95% CI 0.04-0.65). The combination of face-to-face and video calls was most strongly associated with a higher level of perceived family well-being (beta=0.81, 95% CI 0.45-1.16).

**Conclusions:**

The differential use of ICTs to share family life information was observed. The prevalence of video calls was low, but associated with much better family well-being. The results need to be confirmed by prospective and intervention studies to promote the use of video calls to communicate and share information with family, particularly in disadvantaged groups.

## Introduction

Family life information refers to information that strengthens family functioning through improving communication skills, knowledge about developmental tasks, decision-making skills, self-esteem, and interpersonal relationships [1]. Previous studies on family life information have focused on specific topics such as parenting, childcare, and partner relationships [2,3]. The authors adopted a broad, simple, and practical definition of family life information related to family communication, relationships with family members such as children and partner, family activities, work-life balance, and emotion and stress management because these components are reported as main factors affecting family well-being [4,5]. Sharing family life information is a reflection of obtaining information (intentional seeking and unintentional exposure), trust, and perceived usefulness. The authors have reported that these factors were associated with better family well-being [6]. Sharing information can prompt conversation and may facilitate positive family communication, which is crucial for family well-being [7]. Family well-being, often conceptualized as “family life satisfaction,” “sense of well-being,” and “family function” [8], is an important embodiment of collectivism culture, where cohesion and harmony among family members, dependence on the family, and strict obedience of parents are favored [9,10]. Family health, happiness, and harmony (3Hs) were perceived to be significant for families in Hong Kong [11,12].

With advances in technology and high prevalence of mobile phone ownership and Internet penetration, the use of information and communication technologies (ICTs), including instant messaging (IM), social media sites, video calls, and email, to share information is increasing dramatically. These newly emerging ICTs enable people to communicate and share information more conveniently, interactively, and at lower cost. For instance, IM enables users to send information by text, photograph, audio clips, and video at any time, and can reach many individuals simultaneously. Social media sites allow for interconnectivity and provide a platform for information sharing. Video calls provide visual cues along with immediate interaction and feedback for geographically separated individuals [13].

Hong Kong is the most modernized and westernized city in China. There is also widespread penetration of mobile phones and the Internet (in 2015, approximately 83.3% and 84.3% of adults had used a mobile phone and the Internet in the past 12 months, respectively) owing to the advanced cyber-infrastructure and low cost of access to the Internet [14]. Mobile phone ownership and Internet connection in Hong Kong are among the most prevalent in the world [15,16]. Hong Kong has a wide coverage of free public Wi-Fi services (>44,000 hotspots in 2017) [17]. The Internet connection speed in Hong Kong is also among the highest in the world (second in 2015) [18]. Hong Kong has the highest number of young people reporting daily or greater Internet use (68%) compared to other Asian countries [19]. The prevalence of Internet addiction and problematic Internet use were 3% and 31.6%, respectively [19].

However, people with low socioeconomic position (SEP) (lower education or income) often have low access and usage of ICTs [20,21], which may be attributed to the differences among social groups in their ability to access, process, and act on information (communication inequality theory) [22]. For instance, the prevalence of personal computers at home with Internet connection for people with a monthly household income less than HK $10,000 (US $1=HK $7.80) is much lower than those with more than HK $50,000 (35.6% vs 97.9%) [14]. The authors previously proposed the “Inverse ICT Law” [23] based on the Inverse Information Law, which states that the access to appropriate information is particularly difficult for those who are most in need [24,25]. Based on the Inverse ICT Law, those who are most in need may have less access to family-related information, services, and care communicated by ICTs. Online family life information seeking is socially patterned, with lower SEP associated with lower frequency of seeking and paying attention to such information [6,26]. However, people with low SEP also have greater needs to improve their family relationship and family well-being [27].

Despite the high prevalence of ICT use, traditional communication methods (face-to-face and phone) are most used in a family context in Hong Kong, along with a higher level of family well-being [28]. Face-to-face communication includes verbal, nonverbal, and social context cues with real-time feedback and interaction, which can provide greater communication satisfaction [29,30]. Phone calls enable people separated by a long distance to communicate with immediate feedback and real-time interaction. The use of ICTs, including IM, social media, and email, for family communication is not associated with a higher level of family well-being because of the disconnection between verbal and nonverbal signals, impacting the quality of communication [28]. However, the use of video calls may act as a good alternative when face-to-face communication is not possible. Some studies have found that ICTs can strengthen family bonds and improve family cohesion through sharing online activities among family members, such as watching movies and co-playing video games [31-33]. Nevertheless, excessive use of ICTs may reduce time with family and create intergenerational conflicts [34,35], and is associated with poor family relationships [36,37].

To the best of the authors’ knowledge, no studies have investigated the use of different methods to share family life information or its association with family well-being. The authors used a large population-based telephone survey to investigate the pattern and social determinants of family life information sharing with family and the associations of different methods of family life information sharing, especially video calls, with perceived family well-being in Chinese adults living in Hong Kong. The authors examined whether the findings support the Inverse ICT Law on family information sharing.

## Methods

### Study Design

The Hong Kong Family and Health Information Trends Survey (FHInTs) was part of a project entitled “FAMILY: A Jockey Club Initiative for a Harmonious Society.” FHInTs was a regular periodic population-based telephone survey of the general Hong Kong public’s opinions and behaviors on family health, information use, and health communication. Since 2009, five waves of FHInTs have been conducted and details are reported elsewhere [6,26]. The most recent wave was conducted from January to August 2016 to collect data on ICT use on family and health information, family communication, and family well-being.

All interviews were conducted by trained interviewers of the Public Opinion Program at The University of Hong Kong using a Web-based computer-assisted telephone interview system. The survey targeted the Cantonese-speaking adult population aged 18 years and older. Hong Kong residents aged 18 or older were eligible to participate in the telephone survey. Respondents who were psychologically or physically unable to communicate or were unable to communicate using Cantonese over the phone were excluded. Landline telephone numbers were randomly generated using known prefixes assigned to telecommunication services providers under the Numbering Plan provided by the government Office of the Communications Authority. When contact was successfully established with a target household, one qualified person was selected from all those present using the “next birthday” rule [38]. The person from the household who had the nearest next birthday among all household members who were aged 18 years and older was interviewed. Verbal informed consent was obtained from the respondents. Ethical approval was granted by the Institutional Review Board of the University of Hong Kong / Hospital Authority Hong Kong West Cluster.

The most recent wave consisted of four subsets: health, health information, family information, and family communication. Each subset had core questions (questions in all subsets) and subset-specific questions. The authors set the sampling error at 3.1% with 5% type I error. Based on the population size in mid-2009 (N=6,143,300) [39], the authors expected to obtain 1000 successful respondents in each subset. Eligible respondents were randomly assigned into these four subsets. Subsets with questions on family life information sharing (family information and family communication) are included in this analysis (N=2017).

### Measurements

Definitions of family (family members who are related through biological, marital, cohabitation, and/or emotional bonding) and family life information (as mentioned previously) were explained to the respondents before asking questions about family life information sharing and family 3Hs. Methods of family life information sharing were assessed by asking respondents the usual methods of sharing family life information with their family, including face-to-face, phone, IM, social media sites, video calls, and email. Family 3Hs were measured by using three separate questions with a score from 0 to 10. Family well-being was calculated based on the composite score of the 3Hs with higher scores indicating better family well-being. In this sample, the Cronbach alpha coefficient of family well-being was .89, indicating good internal consistency [40].

Socioeconomic position was measured using educational attainment, employment status, and monthly household income. Educational attainment was categorized as primary or below, secondary, and tertiary or above. Employment status was categorized as full time, part time, self-employed, and unemployed. Monthly household income was categorized as <HK $10,000, HK $10,000-$19,999, HK $20,000-$29,999, HK $30,000-$39,999, and >HK $40,000.

### Statistical Analysis

To improve the representativeness of the findings, the raw data were weighted using the random iterative method [41,42] according to provisional figures obtained from the Census and Statistics Department on the gender-age distribution of the Hong Kong population at the end of 2015 and the educational attainment (highest level attended) distribution in the 2011 census. Poisson regression models with robust variance estimators [43] yielded adjusted prevalence ratios (aPR) of different methods of family life information sharing related to age, gender, marital status, and SEP. Multivariable linear regression was used to assess the adjusted associations of different methods of family life information sharing with perceived family 3Hs and well-being scores (continuous variables), adjusting for potential confounders including age, gender, educational attainment, employment status, monthly household income, and marital status. All analyses were conducted using STATA version 13.0. A *P* value <.05 was considered statistically significant.

## Results

Of 2017 respondents after weighting, most were women, aged 25 to 64 years, and married or cohabitating (Table 1). Most respondents had secondary or higher education and had monthly household income of HK $30,000 or greater (median monthly income in Hong Kong was HK $25,000 in 2016). Most respondents (79.41%, 1602/2017) had ever shared family life information with their family.

In the total sample after weighting, the most common method of family life information sharing was face-to-face (74.45%, 1502/2017), followed by IM, phone, and social media sites (Table 2). Only a small percentage of respondents shared family life information by video calls (5.89%, 119/2017) and email (5.48%, 111/2017). The use of the face-to-face method was positively related to the use of each of the other methods (all *P*<.001).

**Table 1:** Sociodemographic characteristics of sample (N=2017).

Demographics		Unweighted, n (%)	Weighted, n (%)
**Gender**			
	Men	751 (37.23)	910 (45.11)
	Women	1266 (62.77)	1107 (54.89)
**Age**			
	18-24	245 (12.15)	191 (9.47)
	25-44	371 (18.39)	715 (35.44)
	45-64	773 (38.32)	743 (36.86)
	≥65	628 (31.14)	368 (18.23)
**Marital status**			
	Single	497 (24.64)	584 (28.93)
	Married or cohabitated	1237 (61.33)	1227 (60.86)
	Widowed or divorced	283 (14.03)	206 (10.21)
**Education attainment**			
	Primary or below	468 (23.20)	477 (23.66)
	Secondary	858 (42.54)	970 (48.09)
	Tertiary or above	691 (34.26)	570 (28.25)
**Employment status**			
	Full time	563 (27.91)	763 (37.81)
	Part time	165 (8.18)	190 (9.42)
	Self-employed	68 (3.37)	90 (4.47)
	Unemployed	1221 (60.54)	974 (48.31)
**Monthly household income (HK$)**^a^			
	<10,000	472 (26.27)	368 (20.47)
	10,000-19,999	303 (16.86)	345 (19.17)
	20,000-29,999	292 (16.25)	326 (18.15)
	30,000-39,999	222 (12.35)	236 (13.15)
	≥40,000	508 (28.27)	522 (29.06)
**Family life information sharing**			
	Yes	1563 (77.49)	1602 (79.41)
	No	454 (22.51)	415 (20.59)

^a^US $1=HK $7.80.

**Table 2:** Prevalence (weighted) of different methods of sharing family life information for total sample (N=2017).

Means		Prevalence, n (%)
No sharing		415 (20.59)
**One or more methods**		
	Face-to-face	1502 (74.45)
	Instant messaging	824 (40.86)
	Phone	567 (28.10)
	Social media sites	240 (11.91)
	Video calls	119 (5.89)
	Email	111 (5.48)

**Table 3:** Association of sociodemographic characteristics with the use of different methods to share family life information with family (N=2017).^a^

Sociodemographic characteristic		Method of sharing family life information, aPR (95% CI)^b^						
		Any (at least one) (n=1602)	Face-to-face (n=1502)	Instant messaging (n=824)	Phone (n=567)	Social media (n=240)	Video calls (n=119)	Email (n=111)
**Gender**								
	Men	1	1	1	1	1	1	1
	Women	1.07 (1.02, 1.13)	1.05 (0.99, 1.12)	1.34 (1.19, 1.51)	1.31 (1.11, 1.55)	1.66 (1.19, 2.30)	1.58 (1.04, 2.39)	1.51 (1.03, 2.21)
**Age**								
	18-24	1	1	1	1	1	1	1
	25-44	0.95 (0.88, 1.04)	0.95 (0.86, 1.05)	0.99 (0.80, 1.24)	1.16 (0.80, 1.68)	1.54 (0.81, 2.94)	1.12 (0.50, 2.50)	1.80 (0.40, 8.08)
	45-64	0.88 (0.80, 0.97)	0.89 (0.80, 1.00)	0.87 (0.68, 1.10)	1.06 (0.72, 1.56)	0.96 (0.49, 1.87)	1.17 (0.54, 2.53)	4.13 (0.89, 19.1)
	≥65	0.75 (0.66, 0.84)	0.77 (0.67, 0.88)	0.48 (0.35, 0.64)	0.96 (0.64, 1.44)	0.48 (0.22, 1.06)	0.98 (0.39, 2.45)	3.34 (0.67, 16.6)
	*P* for trend	<.001	<.001	<.001	.42	.002	.89	.06
**Educational attainment**								
	Primary or below	1	1	1	1	1	1	1
	Secondary	1.22 (1.11, 1.34)	1.20 (1.09, 1.34)	2.14 (1.66, 2.76)	1.32 (1.07, 1.63)	2.45 (1.34, 4.49)	4.04 (1.73, 9.47)	2.54 (1.36, 4.75)
	Tertiary or above	1.25 (1.13, 1.38)	1.22 (1.09, 1.36)	2.55 (1.94, 3.34)	1.24 (0.95, 1.60)	3.32 (1.69, 6.51)	5.61 (2.29, 13.74)	4.67 (2.43, 9.01)
	*P* for trend	<.001	.001	<.001	.13	<.001	<.001	<.001
**Employment status**								
	Full time	1	1	1	1	1	1	1
	Part time	0.99 (0.91, 1.07)	1.02 (0.93, 1.12)	1.12 (0.94, 1.33)	1.01 (0.75, 1.34)	1.17 (0.74, 1.86)	1.33 (0.65, 2.69)	1.91 (1.05, 3.49)
	Self-employed	1.03 (0.94, 1.14)	0.97 (0.85, 1.11)	1.01 (0.77, 1.32)	1.10 (0.74, 1.65)	0.92 (0.45, 1.88)	1.87 (0.82, 4.29)	2.58 (1.28, 5.22)
	Unemployed	1.00 (0.94, 1.06)	1.00 (0.94, 1.07)	0.99 (0.87, 1.13)	0.97 (0.79, 1.19)	0.97 (0.68, 1.39)	1.52 (0.94, 2.46)	1.39 (0.87, 2.23)
**Monthly household income (HK$)**^c^								
	<10,000	1	1	1	1	1	1	1
	10,000-19,999	0.99 (0.89, 1.10)	1.02 (0.90, 1.14)	1.15 (0.90, 1.46)	0.84 (0.66, 1.06)	0.75 (0.44, 1.29)	0.80 (0.40, 1.61)	1.01 (0.51, 2.00)
	20,000-29,999	1.07 (0.97, 1.18)	1.10 (0.98, 1.23)	1.39 (1.10, 1.75)	0.82 (0.64, 1.06)	1.12 (0.66, 1.92)	1.06 (0.55, 2.05)	1.15 (0.58, 2.29)
	30,000-39,999	1.16 (1.06, 1.27)	1.23 (1.11, 1.37)	1.54 (1.21, 1.95)	0.85 (0.65, 1.12)	1.11 (0.63, 1.95)	1.50 (0.79, 2.86)	2.38 (1.25, 4.54)
	≥40,000	1.13 (1.03, 1.23)	1.22 (1.10, 1.35)	1.29 (1.02, 1.62)	0.78 (0.61, 0.99)	0.89 (0.52, 1.52)	0.86 (0.44, 1.67)	1.10 (0.57, 2.13)
	*P* for trend	<.001	<.001	.02	.11	.98	.92	.29
**Marital status**								
	Single	1	1		1	1	1	1
	Married/cohabitated	1.11 (1.03, 1.20)	1.08 (0.99, 1.17)	1.46 (1.23, 1.74)	1.39 (1.06, 1.82)	2.07 (1.34, 3.19)	1.49 (0.84, 2.64)	2.79 (1.14, 6.85)
	Others	1.03 (0.91, 1.17)	0.94 (0.81, 1.09)	1.50 (1.13, 1.99)	1.49 (1.07, 2.08)	1.75 (0.86, 3.56)	0.90 (0.36, 2.26)	2.40 (0.84, 6.85)

^a^All variables were mutually adjusted.

^b^aPR: adjusted prevalence ratio.

^c^US $1=HK $7.80.

More women shared family life information than men by all methods except face-to-face (all *P*<.05) (Table 3). Younger age was associated with the use of any method, face-to-face, IM, and social media sites (all *P* for trend <.01). Higher education level was associated with the use of any method, face-to-face, IM, social media sites, video calls, and email (all *P* for trend <.01), with the strongest association observed for video calls (aPR=5.61, 95% CI 2.29-13.74). Higher monthly household income was associated with the use of any method, face-to-face, and IM to share family life information (all *P* for trend <.05). However, household income was inversely associated with the use of phone, particularly for respondents with household income higher than HK $40,000 (aPR=0.78, 95% CI 0.61-0.99).

Family life information sharing by any method and face-to-face were strongly associated with higher levels of perceived family health, happiness, harmony, and overall family well-being (all beta >0.50) (Table 4). The use of video calls was associated with higher levels of perceived family health (beta=0.36, 95% CI 0.01-0.70), happiness (beta=0.37, 95% CI 0.03-0.70), and overall family well-being (beta=0.34, 95% CI 0.04-0.65). The associations of using IM, social media sites, and email with perceived family 3Hs and well-being were positive but nonsignificant.

**Table 4:** The use of different methods to share family life information with family and perceived family 3Hs and well-being (N=2007).^a^

Method of sharing		Family health		Family happiness			Family harmony			Family well-being		
		Mean (SD)	Beta (95% CI)^b^		Mean (SD)	Beta (95% CI)^b^		Mean (SD)	Beta (95% CI)^b^		Mean (SD)	Beta (95% CI)^b^
**Any**												
	No	6.9 (2.2)	0		7.2 (2.3)	0		7.3 (2.2)	0		7.1 (2.0)	0
	Yes	7.5 (1.7)	0.54 (0.33, 0.76)		7.7 (1.6)	0.53 (0.32, 0.74)		7.8 (1.6)	0.59 (0.39, 0.79)		7.7 (1.5)	0.56 (0.37, 0.75)
**Face-to-face**												
	No	6.9 (2.2)	0		7.2 (2.2)	0		7.3 (2.1)	0		7.1 (1.8)	0
	Yes	7.6 (1.6)	0.59 (0.39, 0.79)		7.7 (1.6)	0.63 (0.44, 0.82)		7.8 (1.6)	0.63 (0.45, 0.82)		7.7 (1.4)	0.62 (0.45, 0.80)
**Instant messaging**												
	No	7.3 (2.0)	0		7.5 (1.9)	0		7.6 (1.9)	0		7.5 (1.7)	0
	Yes	7.6 (1.6)	0.12 (–0.06, 0.30)		7.7 (1.6)	0.11 (–0.07, 0.29)		7.8 (1.6)	0.07 (–0.10, 0.25)		7.7 (1.4)	0.10 (–0.06, 0.27)
**Phone**												
	No	7.3 (1.9)	0		7.5 (1.9)	0		7.6 (1.8)	0		7.5 (1.7)	0
	Yes	7.6 (1.8)	0.22 (0.03, 0.40)		7.7 (1.7)	0.14 (–0.04, 0.32)		7.8 (1.7)	0.18 (0.01, 0.36)		7.7 (1.5)	0.18 (0.02, 0.34)
**Social media**												
	No	7.4 (1.8)	0		7.6 (1.8)	0		7.7 (1.8)	0		7.5 (1.7)	0
	Yes	7.6 (1.7)	0.05 (–0.23, 0.33)		7.8 (1.6)	0.15 (–0.12, 0.42)		7.9 (1.5)	0.18 (–0.08, 0.45)		7.7 (1.5)	0.13 (–0.11, 0.37)
**Video calls**												
	No	7.4 (1.8)	0		7.6 (1.8)	0		7.7 (1.8)	0		7.5 (1.6)	0
	Yes	7.9 (1.5)	0.36 (0.01, 0.70)		8.0 (1.5)	0.37 (0.03, 0.70)		8.0 (1.5)	0.30 (–0.03, 0.63)		8.0 (1.3)	0.34 (0.04, 0.65)
**Email**												
	No	7.4 (1.9)	0		7.6 (1.8)	0		7.7 (1.8)	0		7.5 (1.6)	0
	Yes	7.9 (1.5)	0.32 (–0.02, 0.66)		7.9 (1.6)	0.26 (–0.07, 0.60)		8.0 (1.4)	0.28 (–0.04, 0.61)		7.9 (1.4)	0.29 (–0.01, 0.59)

^a^Family 3Hs and well-being ranged from 0 to 10, with a higher score indicating better outcome.

^b^Adjusted for gender, age, educational attainment, employment status, monthly household income, and marital status.

**Table 5:** The combination of face-to-face and video calls to share family life information with family and perceived family 3Hs and well-being (N=2007).^a^

Method of sharing	Family health		Family happiness		Family harmony		Family well-being	
	Mean (SD)	Beta (95% CI)^b^	Mean (SD)	Beta (95% CI)^b^	Mean (SD)	Beta (95% CI)^b^	Mean (SD)	Beta (95% CI)^b^
Never (n=408)	6.9 (2.2)	0	7.2 (2.3)	0	7.3 (2.2)	0	7.1 (2.0)	0
Face-to-face only (n=1392)	7.6 (1.7)	0.57 (0.36, 0.79)	7.7 (1.6)	0.57 (0.36, 0.79)	7.8 (1.6)	0.63 (0.42, 0.84)	7.7 (1.5)	0.60 (0.41, 0.79)
Video calls only (n=12)	8.1 (1.6)	0.80 (0.23, 1.82)	8.0 (1.7)	0.59 (, 0.42, 1.60)	8.4 (1.5)	0.85 (–0.13, 1.83)	8.2 (1.5)	0.75 (–0.15, 1.66)
Both (n=107)	7.9 (1.5)	0.80 (0.40, 1.20)	8.0 (1.5)	0.82 (0.43, 1.22)	8.0 (1.5)	0.78 (0.40, 1.16)	8.0 (1.3)	0.81 (0.45, 1.16)
Others^c^ (n=88)	6.8 (1.9)	–0.13 (–0.56, 0.30)	6.8 (1.9)	–0.30 (–0.72, 0.12)	7.1 (2.0)	–0.10 (–0.51, 0.31)	6.9 (1.8)	–0.17 (–0.54, 0.21)

^a^Family 3Hs and well-being ranged from 0 to 10, with a higher score indicating better outcomes.

^b^Adjusted for gender, age, educational attainment, employment status, monthly household income, and marital status.

^c^Other methods included any method except face-to-face and video calls.

Compared with the respondents who had never shared family life information, the use of both face-to-face and video calls appeared to be most strongly associated with higher levels of perceived family health (beta=0.80, 95% CI 0.40-1.20), happiness (beta=0.82, 95% CI 0.43-1.22), harmony (beta=0.78, 95% CI 0.40-1.16), and overall family well-being (beta=0.81, 95% CI 0.45-1.16), although the 95% CIs overlapped with the use of face-to-face only (Table 5).

## Discussion

This study provides the first evidence of family life information sharing in one of the most developed non-Western urban settings with high penetration of mobile phones and Internet, and widespread and fast Internet connection. Although the 95% CIs overlapped, it is noteworthy that respondents with higher education were much more likely to share family life information with family by video calls (aPR=5.61, 95% CI 2.29-13.74). This study also found that family life information sharing by the combination of face-to-face and video calls appeared to be most strongly associated with higher levels of perceived family 3Hs and overall well-being.

This study shows that women in Hong Kong are more likely to share family life information than men are, especially by ICTs. However, researchers showed an emerging trend that both genders have equal access to the Internet in developing Asian countries such as Vietnam [44]. Given that mobile phone ownership and Internet access are more prevalent in men than women in Hong Kong [14], such reverse gender difference in family life information sharing by ICTs may be explained by the gender-specific family orientation in Chinese context. Most men are breadwinners and women take care of the family and are therefore more likely to share family life information.

A previous survey showed that people with low SEP were less likely to seek family life information online and use ICTs to communicate with family members [6,28]. This study adds to the literature by showing that people with low SEP are also less likely to share family life information, particularly by ICTs. Lack of cognitive skills, social support, information literacy, and Internet access are documented barriers [14,45]. Household income was inversely associated with the use of the telephone, indicating that more high-income individuals use ICTs to replace the conventional telephone. Compared with income, education was more strongly associated with the use of ICTs to share family life information, indicating that cognitive skills are more important than physical access to the Internet. Sufficient cognitive skills are necessary to understand the content, evaluate the usefulness, and share with others. The wide coverage of free public Wi-Fi services may reduce the access gap between rich and poor. The strongest association of education with the use of video calls still had a low prevalence of use (<10%), and could add new evidence of the emergence of the Inverse ICT Law. However, it also suggests a great potential to improve family life information sharing by video calls in disadvantaged groups, such as increasing the accessibility of video calls (ie, free of charge), ensuring it is user-friendly, and making people aware of the potential family benefits with increased communication. As the costs for subscription to high-speed data packages for home Wi-Fi are decreasing, it leads to more people abandoning the conventional telephone communication method (and saving money), thus video calls could become more popular and could be a greater benefit to underprivileged families.

Moreover, the authors found that family life information sharing was associated with all three dimensions of family well-being (health, happiness, and harmony). Intervention studies have found that family life education programs have benefits of forming and sustaining healthy relationships and improving family functions because family life information can help manage family activities, cope with family problems effectively, and deliver care of the children and the elderly [46,47]. In addition, sharing such information with family can promote positive communication among family members, which is a characteristic of well-functioning family [48].

Notably, the authors found that the use of face-to-face sharing of family life information was most strongly associated with all three dimensions of family well-being. Previous studies in Hong Kong and elsewhere [30,49,50] have reported that face-to face communication is most commonly endorsed compared to recently emerging ICTs and that using face-to-face communication with family, rather than new ICTs, is associated with better family well-being [28]. This study on family communication focusing on family life information showed similar results. Although frequent use of ICTs is observed particularly in young people, face-to-face remains the main mode of communication and information sharing in a family context [32,33,51,52]. A possible explanation is that face-to-face information sharing delivers verbal, nonverbal, and social context cues simultaneously and receives immediate and synchronized feedback of the information, indicating greater communication satisfaction and better information interpretation [30]. Moreover, the authors found that video calls were associated with a higher level of family well-being. Video calls can act as a good alternative when face-to-face is not possible because they provide visual cues with synchronized interaction and feedback. On the contrary, the use of other ICTs, such as IM, social media, or email, to share information may disconnect verbal and nonverbal signals and constrain the number of cues [35,53], and information is easily missing or misinterpreted.

### Limitations

This study has some limitations. First, the cross-sectional study could have residual confounding or the temporal sequence of family life information sharing and family well-being was uncertain. Second, the methods of family life information sharing were determined only by a simple yes/no question; more detailed information such as the frequency of using face-to-face or ICTs to share should be collected in future research. Nevertheless, the authors have shown that a simple question could yield preliminary data to show the presence of the Inverse ICT Law and guide more in-depth studies. Third, the sampling method only covered adults. However, adolescents are more active digital users and more likely to embrace ICTs in various forms. Exploration of ICT use in young people may enable a better understanding of how it affects family well-being. In addition, online interpersonal influences may affect health-related quality of life in adolescents [44]. Finally, because of the small numbers for some uncommonly used methods, such as video calls, the 95% CIs of the aPRs and beta coefficients overlapped, meaning that differences could be due to chance. A much larger sample size is needed for more detailed subgroup analyses.

### Future Work

This study suggests several avenues for future research. First, qualitative research on family life information sharing should be conducted in this setting for a deeper understanding of information sharing behaviors in a family context. Prospective cohort studies and intervention studies are also needed to assess the impact of family life information sharing by video calls on family well-being. Second, information on specific groups of families is important to further address the impact of ICT use on those with special needs. For instance, in families with members living in geographically separated areas, ICTs such as video calls can be increasingly used to maintain family relationships and bonds [54]. ICTs such as social media can be used to provide support for family members who provide care for other members who suffer from chronic diseases and improve family well-being [55]. However, in families with adolescents living in the same house where face-to-face encounters occur frequently, high frequency of ICTs can lead to negative impact, such as a lower level of family cohesion [34,35] and Internet addiction [19,56]. Third, because previous studies found that mobile phone ownership [19], time management problems (ie, the average time spent on the Internet per day) [56], and psychological well-being [57] might affect ICT use, further research is also warranted to investigate how these factors affect the relationship between ICT use and family well-being.

### Conclusions

Although the impact of ICT usage on family has been extensively studied, this study has provided the first evidence of different methods of information sharing with family, especially video calls, and their associations with family well-being. The differential use of ICTs to share family life information supports the emergence of the Inverse ICT Law. Face-to-face communication remained the main mode for family life information sharing and was associated with better family well-being. The prevalence of video calls was low but associated with better perceived family well-being, denoting a feasible way by better use of ICTs to improve family well-being. Further prospective and intervention studies are warranted to confirm the results and to promote the use of video calls to communicate and share information with family, particularly in disadvantaged groups.

## Abbreviations

aPR: adjusted prevalence ratio
FHInTS: Family and Health Information Trends Survey
ICT: information and communication technology
IM: instant messaging
SEP: socioeconomic position
